# Impact of vaccinating dialysis patients: the three waves of COVID-19 analysis

**DOI:** 10.1080/0886022X.2023.2266227

**Published:** 2023-10-11

**Authors:** Fernanda Salomão Gorayeb-Polacchini, Heloisa Cristina Caldas, Mario Abbud-Filho

**Affiliations:** a Department of Nephrology, School of Medicine of São José do Rio Preto (FAMERP), Hospital de Base de São José do Rio Preto/FUNFARME, São José do Rio Preto, Brazil; b Laboratory of Immunology and Experimental Transplantation (LITEX), Faculdade de Medicina de São José do Rio Preto (FAMERP), São José do Rio Preto, Brazil

Dialysis patients were at high risk of severity from COVID-19 [1,2], despite vaccination against SARS-CoV-2 appears to be effective in this population [3]. The emergence of variants of concern has the potential to significantly alter the trajectory of a disease, primarily due to their high transmissibility and resistance to immunity, often resulting in distinct epidemiological waves of infection [4].

The aim of our study was to assess whether vaccination provides effective protection against both COVID-19 infection and its severity among dialysis patients. To achieve this, we conducted a comparative analysis across three distinct epidemiological waves.

This is a prospective observational study involving the analysis of 583 patients receiving chronic dialysis (hemodialysis and peritoneal dialysis), conducted from May 2020 to April 2022 at a single dialysis center.

As part of our screening process, patients were assessed in the waiting room before their dialysis sessions. This assessment involved a verbal questionnaire to inquire about symptoms related to COVID-19 or any recent contact with individuals who had tested positive for the virus. Additionally, we measured the patients’ body temperature. If a patient exhibited any of these symptoms or signs, we promptly conducted an RT-PCR or antigen test, depending on the method available during the respective period. Patients who tested positive for COVID-19 were subsequently included in our study (Figure S1).

The study period was divided into three distinct waves: the first wave from May 2020 to January 2021; the second wave from February 2021 to October 2021; and the third wave from November 2021 to April 2022. These divisions corresponded to the epidemiological peaks of COVID-19 within the dialysis unit, as illustrated in Figure S2.

The study was approved by the Ethics and Research Committee of FAMERP #4212395.

Categorical variables were analyzed by assessing their frequency, while numerical variables were summarized using the mean and standard deviation (SD). The normality of the distribution was evaluated using the Shapiro–Wilk test. For categorical variables, the Chi-square test or Fisher’s exact test, when appropriate, was employed for examination. Continuous variables were compared either through the Kruskal–Wallis method or one-way ANOVA. All data analyses were carried out using GraphPad Prism 9 Software (La Jolla, CA), with significance denoted by a *p* value <.05.

Of the total of 583 dialysis patients followed during the study period, 225 (38.5%) tested positive for COVID-19, 47/451 in the first wave, 77/420 in the second wave, and 101/374 in the third epidemiological wave of the disease (Figure S1).

Regarding the 225 positive patients, 85.8% were patients on hemodialysis, 29.3% required the use of oxygen, 11.1% mechanical ventilation (MV), 33% hospitalization, 14.7% intensive care unit (ICU) admission, and the case fatality rate was 14.7%. The incidence and mortality rates were calculated per 10,000 exposed patients with results of 3859.3 and 566, respectively (Table 1). Regarding the treatment of COVID-19, only hospitalized patients received corticosteroids and prophylactic anticoagulants, none patients were administered any other COVID-19 specific treatments.

**Table 1.** Comparative analysis of clinical profiles and COVID-19 patient outcomes across three waves.

Features	Total (*n* = 225)	First wave (*n* = 47)	Second wave (*n* = 77)	Third wave (*n* = 101)	*p* Value
Treatment, *n* (%)					
Oxygen	66 (29.3)	18 (38)*	38 (49.3)**	10 (10)	–
Mechanical ventilation	25 (11.1)	6 (12.7)*	18 (23.3)**	1 (1)	–
Hospitalization	74 (33)	21 (41)*	40 (52)**	13 (13)	–
ICU admission	33 (14.7)	11 (27)*	19 (24.6)**	3 (3)	–
Incidence rate/10,000	3859.3	1042.1^#,^*	1833.3**	2700.5	–
Mortality rate/10,000	566	177.3^#^	523.8**	80.2	–
Case fatality rate (%)	33 (14.7)	8 (17)*	22 (29)**	3 (3)	–
Mild disease, *n* (%)	144 (64)	23 (49)*	35 (45.5)**	87 (86)	–
Moderate disease, *n* (%)	45 (20)	13 (27.6)*	14 (18.2)	9 (9)	–
Symptoms, *n* (%)					
Fever	113 (50.2)	32 (68)*	42 (54.5)**	39 (38.6)	–
Cough	137 (61)	24 (51)	47 (61)	66 (65,3)	.25
Dyspnea	76 (33.8)	24 (51)*	35 (45.4)**	17 (16.8)	–
Diarrhea	34 (15)	16 (34)*	13 (16.9)**	5 (5)	–
Asthenia	57 (25.3)	16 (34)*	24 (31.2)**	17 (16.8)	–
Laboratory findings, mean (SD)					
Hemoglobin (g/dL)	11 ± 2	10.4 ± 1.9	11 ± 2	11 ± 2	.15
Platelets per mm^3^	183,443 ± 95,168	178,532 ± 91,883	184,986 ± 121,683	184,761 ± 71,609	.9
Leukocytes per mm	5709 ± 2797	5564 ± 2598	5330 ± 2628	6076.7 ± 2995	.14
Lymphocytes per mm	1088.6 ± 519.3	1107.4 ± 602	909.9 ± 461.8**	1217.6 ± 480	**.0007**
Neutrophils per mm^3^	3827 ± 2457	3753 ± 2350.7	3799 ± 2324	3886 ± 2623	.96
NLR	4.89 ± 7	4.6 ± 5.4	6.3 ± 10**	3.9 ± 3.8	**.04**
CRP (mg/dL)	6.6 ± 8.4	8.6 ± 10.2	7.7 ± 9**	4.56 ± 6	**.01**
SGOT (U/L)	24.2 ± 22	28 ± 35	26 ± 17.6	20.6 ± 14	**.04**
D-dimer (μg/mL)	2.23 ± 2.35	2.4 ± 3	1.8 ± 1.4	2.5 ± 2.4	.32
LDH (U/L)	259.3 ± 100	295 ± 107.3*	286.1 ± 116**	219 ± 6	**<.001**
Reinfection, *n* (%)	17 (7.5)	0	0	17 (16.8)	–

CRP: C-reactive protein; SD: standard deviation; CKD: chronic kidney disease; ICU: intensive care unit; LDH: lactate dehydrogenase; NLR: neutrophil–lymphocyte ratio; SGOT: serum glutamic-oxalacetic transaminase.

Data are presented as mean ± SD for continuous variables and as percentages with absolute numbers for categorical variables. The analyses for comparing the three groups were conducted using ANOVA or Kruskal–Wallis with multiple comparison tests. The significant *p* values are indicated in bold. Calculations: incidence = number of cases/number of people exposed per 10,000. Mortality = number of deaths from COVID-19/number of people exposed per 10,000. Fatality = (number of COVID-19 deaths/number of COVID-19 cases) × 100.

*First wave vs. third wave, *p* < .05.

** Second wave vs. third wave, *p* < .05.

^#^ First wave vs. second wave, *p* < .05.

Demographic characteristics, comorbidities, and the type of dialysis did not exhibit statistically significant differences among the groups. Notably, patients in the third wave displayed fewer symptoms and lower laboratory values for inflammatory markers compared to those in the first and second wave groups. The third epidemiological wave, despite the higher incidence, presented lower severity of the disease, with less mortality and fatality. The fatality rate of the first, second and third waves is 17%, 29%, and 3%, respectively.

The diagnosis of COVID-19 that occurred at least 14 days after the last dose of vaccine was considered. As shown in Table 2, the vaccination rate in the third wave was 100% of patients with at least two doses of COVID-19 vaccine and 96% of patients with three doses of vaccine, while in the second wave only 10.3% of patients were vaccinated with two doses and in the first wave, no patients were vaccinated. Regarding the outcomes in relation to the number of vaccine doses, patients with three doses of vaccine had a lower rate of hospitalization, ICU, MV, and death. The vaccines used for three doses were: 50.4% with two doses of ChAdOx1 + 1 CoronaVac followed by 34.6% of three doses of CoronaVac and 5.9% of two doses of ChAdOx1 + 1 dose of BNT162b2.

**Table 2.** Patient outcomes in relation to the number of vaccine doses.

	No vaccination (*n* = 69)	1 vaccine dose (*n* = 37)	2 vaccine doses (*n* = 22)	3 vaccine doses (*n* = 97)
Hospitalization, *n* (%)	34 (49.2)*	18 (48.6)**	10 (45.4)***	12 (12.3)
Intensive care, *n* (%)	20 (28.9)*	6 (16.2)**	4 (18.1)***	3 (3)
Mechanical ventilation, *n* (%)	13 (18.8)*	5 (13.5)**	6 (27.2)***	1 (1)
Death, *n* (%)	17 (24.6)*	6 (16.2)***	7 (31.8)	3 (3)

Vaccine dose: the diagnosis of COVID-19 occurred at least 14 days after the last dose of vaccine.

* No vaccination vs. three vaccine doses, *p* < .01.

** One vaccine dose vs. three vaccine doses, *p* < .01.

*** Two vaccine doses vs. three vaccine doses, *p* < .01.

The logistic regression analysis shows a higher risk of hospitalization (OR 6.5; CI 3.3–12.94) and death (OR 10.4; CI 3–35.3) for patients in the first and second wave and higher risk of hospitalization (OR 9.7, CI 2.8–33.1), and death (OR 6.0, CI 3–11.9) for patients with less than three doses of vaccine.

In discussion, the patients with COVID-19 in our study had lower severity and fatality of the disease despite the higher incidence in the third epidemiological wave, with higher vaccination rate and number of vaccine doses in this period. The most prevalent symptoms in our study were similar to other dialysis unit studies [5].

In a prior study conducted by the same group, a lower incidence of COVID-19 and a higher fatality rate were observed, which may be attributed to the absence of vaccination during that time [5]. Another multicenter study in Brazil also reported lower incidence and mortality rates but higher fatality rates compared to our present study [2]. These differences are likely influenced by socioeconomic disparities, the timing of the study period, and the varying incidence of the disease in different regions [2].

In another study published from our dialysis center, we compared the first two epidemiological waves of COVID-19, the second wave, more severe, although with a higher percentage of patients with two doses of vaccine, but probably related to change of variant in the period with the prevalence of the *Gamma* variant [6], with similar results in another study [7]. In our current study, we observed a higher fatality rate during the second wave compared to the lower fatality rate seen in the third wave. This difference may be attributed to a higher vaccination rate during the study period and the prevalence of the *Omicron* variant in the third wave [8].

It is worth noting that the *Omicron* variant is characterized by an extensive set of mutations in the spike protein, which gives it a high potential for infectivity and the ability to partially evade the immune response in patients. However, despite its increased transmissibility, this variant exhibits lower virulence, which could explain the higher incidence of cases but a lower fatality rate during the third wave in our study [4].

A review study shows a lower seroconversion after vaccination of dialysis patients in relation to the general population, hence the importance of at least three doses of vaccine in this population [5].

Limitations of our study include the absence of variant sequencing; instead, we relied on the prevailing regional variants during the respective time periods as parameters. Additionally, we were unable to measure SARS-CoV-2 Ig levels due to budgetary constraints.

In conclusion, our study suggests that a three-dose vaccination regimen, along with the prevalence of the *Omicron* variant, may have contributed to a milder disease course among dialysis patients during the third epidemiological wave of COVID-19. Despite a higher incidence of cases, this wave exhibited lower disease severity, mortality and ­fatality rates.

## Supplementary Material

Supplemental Material

Supplemental Material
