# Supplementary material for: Impact of vaccinating dialysis patients: the three waves of COVID-19 analysis
Source: Ren Fail. 2023 Oct 11;45(2):2266227. doi: 10.1080/0886022X.2023.2266227 (PMC10569342; doi:10.1080/0886022X.2023.2266227)
Supplement: Supplemental Material [file IRNF_A_2266227_SM7928.pdf]

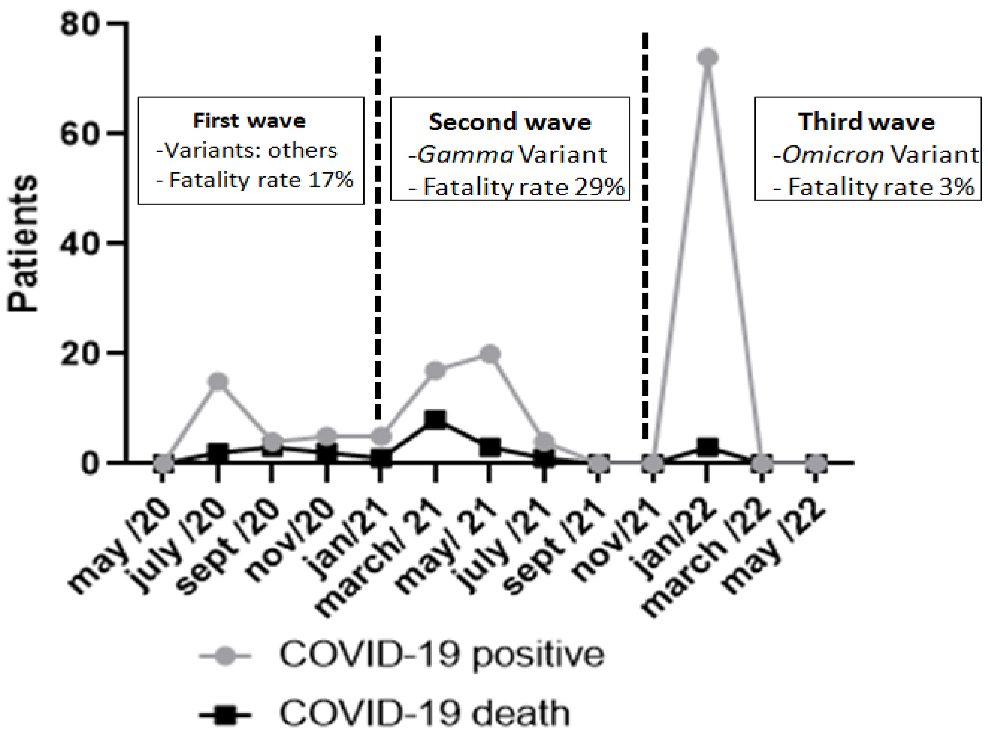

Others variants: B.1.1.33, P2.

Figure S2. Figure the three waves and the variants were more common in that period with the COVID-19 positive and death patients in the periods.

Information Classification: General
