# Supplementary figures and images for: Impact of vaccinating dialysis patients: the three waves of COVID-19 analysis
Source: Ren Fail. 2023 Oct 11;45(2):2266227. doi: 10.1080/0886022X.2023.2266227 (PMC10569342; doi:10.1080/0886022X.2023.2266227)

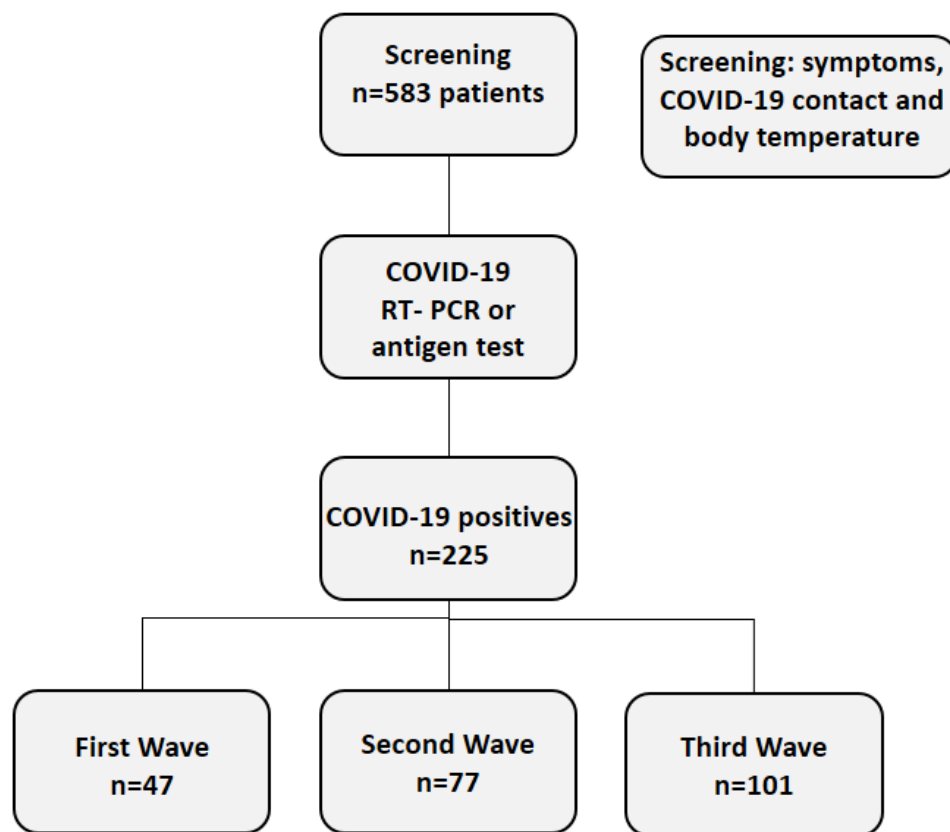

**Figure S1.** Study design. RT-PCR reverse transcription polymerase chain reaction

Supplement: Supplemental Material [file IRNF_A_2266227_SM7923.pdf]
